# Topological transitions among skyrmion- and hedgehog-lattice states in cubic chiral magnets

**DOI:** 10.1038/s41467-019-08985-6

**Published:** 2019-03-05

**Authors:** Y. Fujishiro, N. Kanazawa, T. Nakajima, X. Z. Yu, K. Ohishi, Y. Kawamura, K. Kakurai, T. Arima, H. Mitamura, A. Miyake, K. Akiba, M. Tokunaga, A. Matsuo, K. Kindo, T. Koretsune, R. Arita, Y. Tokura

**Affiliations:** 1 Department of Applied Physics, The University of Tokyo, Bunkyo-ku, Tokyo, 113-8656 Japan; 2 RIKEN Center for Emergent Matter Science (CEMS), Wako, Saitama, 351-0198 Japan; 3 Neutron Science and Technology Center, Comprehensive Research Organization for Science and Society (CROSS), Tokai, Naka, Ibaraki, 319-1106 Japan; 4 Department of Advanced Materials Science, The University of Tokyo, Kashiwa Chiba, 277-8561 Japan; 5 The Institute for Solid State Physics (ISSP), The University of Tokyo, Kashiwa Chiba, 277-8581 Japan; 6 Department of Physics, Tohoku University, Aoba-ku, Sendai, Miyagi 980-8578 Japan

## Abstract

Manipulating topological spin textures is a key for exploring unprecedented emergent electromagnetic phenomena. Whereas switching control of magnetic skyrmions, e.g., the transitions between a skyrmion-lattice phase and conventional magnetic orders, is intensively studied towards development of future memory device concepts, transitions among spin textures with different topological orders remain largely unexplored. Here we develop a series of chiral magnets MnSi_1−*x*_Ge_*x*_, serving as a platform for transitions among skyrmion- and hedgehog-lattice states. By neutron scattering, Lorentz transmission electron microscopy and high-field transport measurements, we observe three different topological spin textures with variation of the lattice constant controlled by Si/Ge substitution: two-dimensional skyrmion lattice in *x* = 0–0.25 and two distinct three-dimensional hedgehog lattices in *x* = 0.3–0.6 and *x* = 0.7–1. The emergence of various topological spin states in the chemical-pressure-controlled materials suggests a new route for direct manipulation of the spin-texture topology by facile mechanical methods.

## Abstract (web summary)

Manipulating topological spin textures are demanded for future spintronic devices, but knowledge about phase transitions among different spin textures remain limited. Here, Fujishiro and Kanazawa et al. report chemical-pressure-controlled phase transitions between different topological spin textures in chiral magnets MnSi_1−*x*_Ge_*x*_.

## Introduction

The concept of topology provides a powerful scheme for the classification of electronic and magnetic states, and also for the description of their physical properties^1^. Topology of a magnetic structure is characterized by the winding number $w = \frac{1}{{8{\mathrm{\pi }}}}\epsilon ^{ijk}{\kern 1pt} {\int}_S {{\kern 1pt} dS_k{\mathbf{n}}({\mathbf{r}}) \cdot [\partial _i{\mathbf{n}}({\mathbf{r}}) \times \partial _j{\mathbf{n}}({\mathbf{r}})]}$. This quantity counts how many times the direction of the local magnetization, i.e., **n**(**r**) = **m**(**r**)/|**m**(**r**)|, wraps the unit sphere within the unit area *S*. When a magnetic structure possesses a non-zero integer winding number, it behaves as a topologically stable spin-object, producing emergent phenomena unique to its topological class.

**Fig. 1:**
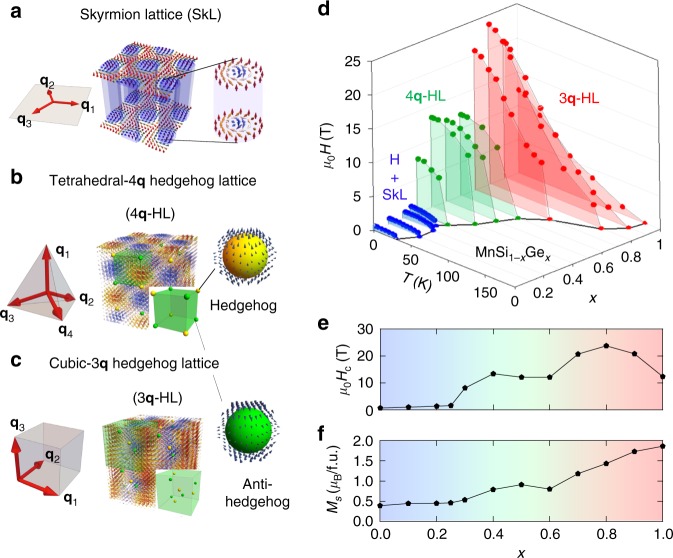
Schematics of topological multiple-*q* spin textures and two-step magnetic transitions in MnSi_1−*x*_Ge_*x*_. **a** Spin configurations of two-dimensional skyrmion lattice (2D SkL), which is a hexagonal array of skyrmion cylinders. It is characterized by three helical modulation vectors $\left( {{\mathbf{q}}_1 + {\mathbf{q}}_2 + {\mathbf{q}}_3 = 0} \right)$ perpendicular to external magnetic field **H**. **b** Spin configurations of tetrahedral-4**q** hedgehog lattice (4**q**-HL), which is described by four **q**-vectors pointing in the apical directions of a regular tetrahedron. Those **q**-vectors are fixed along 〈111〉 crystal axes at zero *H*. Hedgehogs and anti-hedgehogs are at face-centered-cubic positions as illustrated in the bottom right green box representing the magnetic unit cell. **c** Spin configurations of cubic-3**q** hedgehog lattice (3**q**-HL), which is described by three orthogonal **q**-vectors, which are pinned along 〈100〉 crystal axes at zero *H*. In **a**–**c** the red (blue) arrows drawn in each spin texture represent up (down) magnetic moments. **d** Variation of phase boundary between ferromagnetic and helimagnetically ordered states as a function of magnetic field *H*, temperature *T*, and *x* in MnSi_1−*x*_Ge_*x*_. Red, green, and blue color denote 3**q**-HL, 4**q**-HL, and helical(H)/SkL states, respectively. The variation of magnetic transition temperature is indicated by the black line on the bottom plane. (See also Supplementary Fig. 2) **e**, **f** Composition *x* dependence of ferromagnetic transition field *H*_c_ (**e**) and saturation magnetization *M*_s_ (**f**)

A representative example is the magnetic skyrmion, which is a two-dimensional (2D) nanometric vortex-like structure consisting of many electron spins^2–4^. In the bulky compounds, skyrmions elongate in cylindrical forms, usually assembling in a hexagonal lattice, i.e., the skyrmion lattice (SkL). In reciprocal space, the hexagonal SkL can be approximately described as a superposition of three helical modulations with the wavevectors (**q***-*vectors) forming a mutual angle of 120° in a plane perpendicular to external magnetic field *H*, as shown in Fig. 1a. Skyrmion-hosting materials are of great variety, including non-centrosymmetric bulk magnets^5^ and multilayered thin films^6,7^. Through the interaction with conduction electrons, skyrmions generate the effective magnetic field confined in each interior, so-called emergent magnetic field as defined by the Berry curvature $b_k = \frac{1}{2}{\it{\epsilon }}^{ijk}{\mathbf{n}}\left( {\mathbf{r}} \right) \cdot \left[ {\partial _i{\mathbf{n}}\left( {\mathbf{r}} \right) \times \partial _j{\mathbf{n}}\left( {\mathbf{r}} \right)} \right]$. Owing to their topology, skyrmions carry the quantized emergent flux $\phi _0 = - \frac{h}{e}$ in case of the strong spin-charge coupling, which offers attractive spintronic functionalities conserved even in nano-scale devices;^8–10^ such as topological Hall effects^11–13^ and emergent electromagnetic inductions^1,14–16^.

Also as a result of the geometric constraint, transitions of topological spin textures are accompanied by dynamics of topological spin defects, as exemplified by the emergence of spin hedgehogs in the course of creation or annihilation of skyrmions^17,18^. Because transformations between skyrmions and conventional magnetic orders need discrete changes in the winding number, they cannot be realized by smooth change of the directions of local spins. Instead, it is necessary to introduce hedgehog point defects, which are three-dimensional (3D) topological spin structures, behaving as emergent magnetic monopoles or anti-monopoles with non-zero divergence of emergent magnetic field $\left( {\frac{1}{{4{\mathrm{\pi }}}}\nabla \cdot {\mathbf{b}} = \pm 1} \right)$^19^. The motions of hedgehogs locally give or remove the topological winding number, causing the topological transition through the elongation, contraction, coalescence, and division of skyrmion strings^17,18^. In association with such dynamics of topological charges, the topological transition of a spin texture often involves non-trivial emergent phenomena, e.g., the formation of fluctuating topological magnetic order^20^, the concomitant non-Fermi-liquid like behavior^21^ and electrical magnetochiral effect^22^. Spin hedgehogs are also found as a dense lattice form in MnGe, namely the array of hedgehogs and anti-hedgehogs connected by skyrmion strings. This state can be described approximately by three helical modulations with their **q**-vectors forming the orthogonal sides of a cube^23^ and is here referred to as cubic-3**q** hedgehog lattice (HL) (Fig. 1c). The transition from the HL state to the non-topological state, e.g., single-**q** conical and ferromangnetic state, undergoes the pair annihilation of hedgehogs and anti-hedgehogs^24^, which entails critical anomalies of resistivity, elastic property, and thermopower as well^24,25^.

In order to harness the topological properties unique to each spin texture and to explore non-trivial emergent phenomena at their transitions, direct control of topology of spin texture is essential. In this context, switching of the spin textures among plural different topologically nontrivial classes has remained a challenge. We focus on chemical/mechanical pressure as one potential approach to this end, by achieving dramatic modification in magnetic interactions through changing inter-atomic distances.

Here we report on the topological transitions among 2D SkL and 3D HLs in cubic chiral magnets MnSi_1−*x*_Ge_*x*_. By changing chemical pressure through substitution between Si and Ge, in other words, by controlling the lattice constant *a* (Supplementary Fig. 1), we observed that the SkL in MnSi undergoes two-step transitions to the cubic-3**q** HL in MnGe. In the intermediate composition range, we unveiled a new topological spin texture characterized by four **q**-vectors pointing in the apical directions of a regular tetrahedron. This state corresponds to the face-centered-cubic array of the hedgehogs and anti-hedgehogs, which we call tetrahedral-4**q** HL (Fig. 1b). Our neutron scattering experiment and Lorentz transmission electron microscopy (LTEM) observation confirm the conventional SkL for Si-rich composition range (*x* = 0–0.25), tetrahedral-4**q** HL for the intermediate range (*x* = 0.3–0.6), and cubic-3**q** HL for Ge-rich range (*x* = 0.7–1). Furthermore, by high-field Hall resistivity measurements, we identified topological Hall effect of Berry-phase origin in each magnetic phase, supporting their topological spin arrangements.

## Results

### Overview of topological magnetic transitions in MnSi_1−*x*_Ge_*x*_

To overview the magnetic transitions in MnSi_1−*x*_Ge_*x*_, we first show the magnetic phase diagrams for varying *x* as summarized in Fig. 1d. (See Supplementary Fig. 2 for magnetic transition temperatures *T*_N_ for varying *x* in detail.) The drawn phase boundary line for each *x* represent the ferromagnetic transition, determined from magnetization measurements. (See Supplementary Fig. 3 for details.) The variation in the phase diagram indicates that MnSi_1−*x*_Ge_*x*_ can be sorted into three categories, as evident from the distinct value of ferromagnetic-transition magnetic field (*H*_c_) at the lowest temperature; two steep changes in *H*_c_ at *x* ~ 0.3 and *x* ~ 0.7 (*ΔH*_c_ ~ 10 T) suggests the magnetic-structure transitions (Fig. 1e). There are also recognized characteristic profiles in *x*-dependence of other magnetic properties. As for the saturation magnetization (*M*_s_), a plateau-like structure appears around *M*_s_ ~ 1 *μ*_B_/f.u. within the range *x* = 0.4–0.6 (Fig. 1f). Incidentally, the similar feature was observed in a former study on hydrostatic-pressure dependence of *M*_s_ in MnGe^26^.

### SANS and LTEM studies at zero magnetic field

**Fig. 2:**
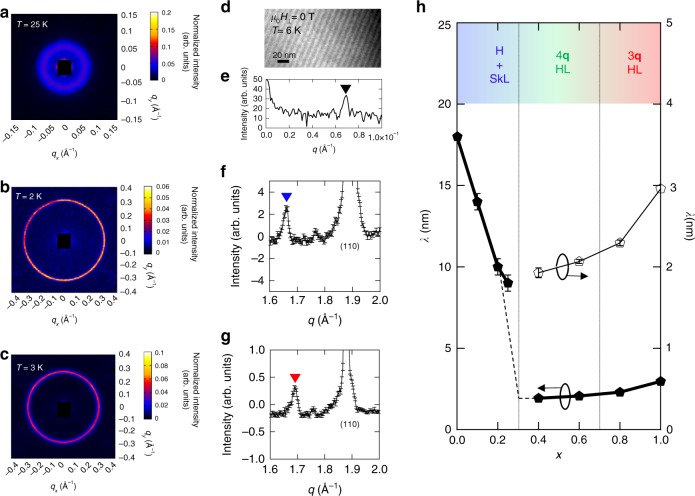
Magnetic modulation periods and directions in MnSi_1−*x*_Ge_*x*_ at zero magnetic field. **a**–**c** The small-angle neutron scattering (SANS) intensity patterns of *x* = 0.2 (*T* = 25 K) (**a**), *x* = 0.6 (*T* = 2 K) (**b**), and *x* = 0.8 (*T* = 3 K) (**c**) after zero field cooling. **d** Lorentz transmission electron microscopy (LTEM) image in (001) crystal plane for *x* = 0.2 (*T* = 6 K), where the helical structure with its **q**-vector running in one of 〈100〉 directions is observed. **e** Fourier transform profile of the LTEM image shown in **d**, with the black triangle representing the peak position (|**q**| ~ 0.069 Å^−1^). **f**, **g** Powder neutron diffraction patterns around the (110) nuclear reflection of *x* = 0.6 (*T* = 2 K) (**f**) and *x* = 0.8 (*T* = 3 K) (**g**). Background signals measured at high temperatures are subtracted. Each satellite peak, which is indicated by blue or red triangle, is assigned to the formation of magnetic structure modulating along 〈111〉 (**f**) or 〈100〉 directions (**g**), respectively. The error bars represent statistical error of one standard deviation. **h** Variation of magnetic modulation period (*λ*) as a function of composition *x* at the lowest temperature (solid markers and lines), which are revealed by LTEM observation (*x* = 0.1, 0.2, and 0.25) and SANS measurement (*x* ≥ 0.4). The open markers for the right axis show the magnified data of *x* ≥ 0.4. The dashed lines show the guide to the eyes for the extrapolated variation of magnetic period in 0.2 ≤ *x* ≤ 0.4. The data of MnSi and MnGe are cited from refs. ^3,13^, respectively. The error bars for *x* ≤ 0.25 are defined by the spatial resolution of Lorentz transmission electron microscope, while those for *x* ≥ 0.4 represent statistical error of one standard deviation of the SANS measurements

In order to clarify the variation of magnetic period (*λ*) and direction of wavevectors (**q**-vectors) in MnSi_1−*x*_Ge_*x*_, small- and wide-angle neutron scattering experiments as well as LTEM observations were performed at zero magnetic field. Figure 2a–c show the SANS intensity patterns of *x* = 0.2, 0.6, and 0.8 after zero field cooling from room temperature. The observed Debye-ring-like patterns indicate the formation of periodically modulated magnetic structures. Their modulation periods are determined from the radius (*q*) of the each diffracted ring pattern as *λ* = 2π/*q* (Fig. 2h).

The **q**-vectors, which are fixed along specific crystal-axes due to magnetic anisotropy, were identified by using LTEM for *x* = 0.2 (Fig. 2d, e) and by wide-angle neutron scattering for *x* = 0.4, 0.6, and 0.8 (Fig. 2f, g). (See also Supplementary Fig. 6 for all the data sets.) In a thin plate sample of *x* = 0.2, there observed a helical structure with **q** || 〈100〉 and *λ* = 9 nm (Fig. 2e). Here we note that the observed **q**-direction is not necessarily the same in bulk samples as seen in the LTEM study on MnSi, where the helical direction is dependent on the crystalline orientation due to large magnetic anisotropy effects at the surfaces^27^. Since the **q**-directions in bulks of MnSi and *x* = 0.4 (Supplementary Fig. 6b) are both parallel to 〈111〉, we speculate that it is also the same in the *x* = 0.2 bulk sample, although the direct verification by wide-angle neutron scattering is difficult within the current *q*-resolution. (See Supplementary Fig. 6a). In powder samples of *x* = 0.6 and 0.8, we show the wide-angle neutron diffraction profiles around (110) nuclear reflection in Fig. 2f, g. Because magnetic reflections generally appear as satellite peaks at the wavenumber of $\left| {{\mathbf{q}}_{\mathrm{n}} \pm {\mathbf{q}}} \right|$ in a powder neutron diffraction, we can determine the ***q***-direction from the satellite peak position once we identify the magnitude of **q** by SANS^13^. Here **q**_n_ is the reciprocal lattice vector. Each satellite peak observed in *x* = 0.6 and 0.8 (indicated by blue and red triangles in Fig. 2f, g) can be indexed as $\left( {\frac{{2{\mathrm{\pi }}}}{a} - \frac{q}{{\sqrt 3 }},\frac{{2{\mathrm{\pi }}}}{a} - \frac{q}{{\sqrt 3 }}, \pm \frac{q}{{\sqrt 3 }}} \right)$ and $\left( {\frac{{2{\mathrm{\pi }}}}{a} - q,\frac{{2{\mathrm{\pi }}}}{a},0} \right)$, respectively. Namely, the **q**-vector directions are along 〈111〉 crystal axes for *x* = 0.6, and 〈100〉 crystal axes for *x* = 0.8.

As summarized in Fig. 2h, there are two features in *x*-dependence of the magnetic modulation **q**; the two-step magnetic transitions observed as distinct changes in the phase diagram (Fig. 1d). One is the discontinuous variation in *λ* (=2π/*q*) at *x* ~ 0.3 and the other is change in the pinned **q**-direction at *x* ~ 0.7. It is therefore clear that three distinct magnetic phases are realized in MnSi_1−*x*_Ge_*x*_. In later sections, those respectively turn out to be helicl/SkL (*x* = 0–0.25), tetrahedral-4**q** HL (*x* = 0.3–0.6), and cubic-3**q** HL (*x* = 0.7–1).

### Formation mechanisms of shortperiod topological spin textures

It is noteworthy that such remarkable variations in the magnetic properties can be driven essentially by such a lattice-constant change without any change in lattice symmetry. Here we discuss the origin of the dramatic magnetic transitions in MnSi_1−*x*_Ge_*x*_. One important indication we obtain is that the dominant magnetic interactions for the 3D HL states may differ from those for 2D SkL where the competition between ferromagnetic exchange interaction (EXI) $( { \propto J{\mathbf{S}}_i \cdot {\mathbf{S}}_j})$ and Dzyaloshinskii-Moriya interaction (DMI) $( { \propto {\mathbf{D}} \cdot ({\mathbf{S}}_i \times {\mathbf{S}}_j)})$ determines the basic magnetic properties^28^, such as $\lambda \sim a \cdot J{\mathrm{/}}D$ (*a* being the lattice constant) and $H_{\mathrm{c}}\,\sim \,D^2M{\mathrm{/}}J$. In the conventional SkL materials, the magnetic ground state is a long-period helical structure (*λ* typically ranges from 10 to 100 nm) with relatively small $H_{\mathrm{c}}$ (<1 T) since the energy scale is well-separated as $J \gg D$. If the same model could be applied, the extremely short $\lambda$ (1.94–2.80 nm) and large $H_{\mathrm{c}}$ (12–24 T) observed in MnSi_1−*x*_Ge_*x*_ (*x* = 0.4–1) would require much larger DMI even exceeding EXI. The band structure calculations^29^, however, demonstrate a contradictory behavior of DMI in MnSi_1−*x*_Ge_*x*_ to this naive expectation: DMI gets rather smaller with increasing *x*, whereas the calculated values of *M*_s_ are in accord with the observed ones (Supplementary Fig. 4). Thus, the DMI may not be the primary origin of the short-period helical structure in 3D HL. Instead, the magnetic frustration or Ruderman-Kittel-Kasuya-Yosida (RKKY) interaction^30–33^ causing competing ferromagnetic and antiferromagnetic EXIs can be a possible mechanism. Possibly related to such conduction-electron mediated exchange interactions, we found that the strong Hubbard-*U* implemented in the band structure calculation favors a short-period helical structure (Supplementary Fig. 5). Further theoretical studies are desired to clarify the crucial magnetic interaction, which takes over from DMI in the course of enlarging the lattice constant.

### SANS and LTEM studies under magnetic fields

**Fig. 3:**
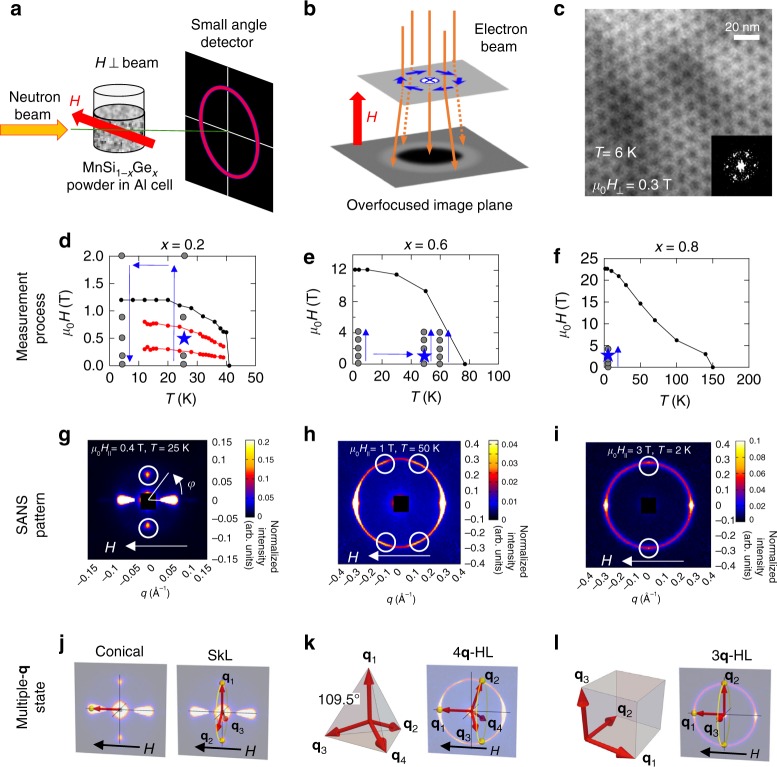
Magnetic structures of MnSi_1−*x*_Ge_*x*_ revealed by small-angle neutron scattering (SANS) and Lorentz transmission electron microscopy (LTEM) under magnetic field. **a** The SANS setup with magnetic field *H* perpendicular to the incident neutron beam. **b** Schematic illustration of LTEM observation with *H* parallel to the incident electron beam (orange lines), where the blue arrows represent the in-plane magnetic moment configuration of a skyrmion. **c** The over-focused LTEM image (*T* = 6 K, *μ*_0_*H* = 0.3 T) in (001) crystal plane and its Fourier transform pattern. **d**–**f** The SANS measurement points (gray dots) and sequences (blue arrows) are shown in the magnetic phase diagrams of *x* = 0.2 (**d**), *x* = 0.6 (**e**), and *x* = 0.8 (**f**), where the blue stars represent the data points shown in **g**–**i**. The SANS intensity patterns of *x* = 0.2 (*T* = 25 K, *μ*_0_*H* = 0.5 T) (**g**), *x* = 0.6 (*T* = 50 K, *μ*_0_*H* = 1 T) (**h**), and *x* = 0.8 (*T* = 2 K, *μ*_0_*H* = 3 T) (**i**). The small white circles emphasize the characteristic peak intensities for each composition. The candidate multiple-q structures explaining the observed SANS intensity patterns are shown in **h**–**l**, where the yellow rings represent the rotation degrees of freedom of **q**-vectors due to randomly oriented crystal domains in the polycrystalline samples and the yellow dots represent the scattering intensities on the detector plane

Having confirmed the variation of magnetic properties in MnSi_1−*x*_Ge_*x*_, we investigated the magnetic structures under magnetic fields by SANS in *x* = 0.2, 0.6, and 0.8 (as representative compositions of the three magnetic phases), and by LTEM in the thin plate of *x* = 0.2. Magnetic field (*H*) is applied perpendicular to the incident neutron beam for SANS experiment (Fig. 3a), while *H* is applied parallel to the electron beam for LTEM measurement (Fig. 3b). As for *x* = 0.2, LTEM directly reveals the formation of a hexagonal SkL in the plane perpendicular to *H*, which is also confirmed by the six-fold Fourier transform image (Fig. 3c).

In Fig. 3d–f, we display all the measurement points (gray dots) and sequences (blue arrows) of SANS in the magnetic phase diagrams, and the representative SANS patterns obtained at the *T*–*H* points (highlighted by blue stars in Fig. 3d–f) are shown in Fig. 3g–i. (See Supplementary Figs 7–9 for the SANS data at other temperatures and magnetic fields). The SANS pattern on the polycrystalline $x = 0.2$ compound (Fig. 3g) exhibits intensity peaks parallel ($\varphi = 0^\circ$, $180^\circ$) and perpendicular ($\varphi = \pm 90^\circ$) to **H**, which indicates the conical state modulating along **H** coexists with SkL. Because only a small portion of SkL states in powder grains can meet the diffraction condition as illustrated in Fig. 3j, the much weaker intensity peaks were observed at $\varphi = \pm 90^\circ$ than those scattered at $\varphi = 0^\circ$, $180^\circ$ by the conical state. Incidentally, we also confirmed that the peaks at $\varphi = \pm 90^\circ$ only emerge in the SkL phase identified by the magnetization measurements. (See Supplementary Fig. 7 for details).

**Fig. 4:**
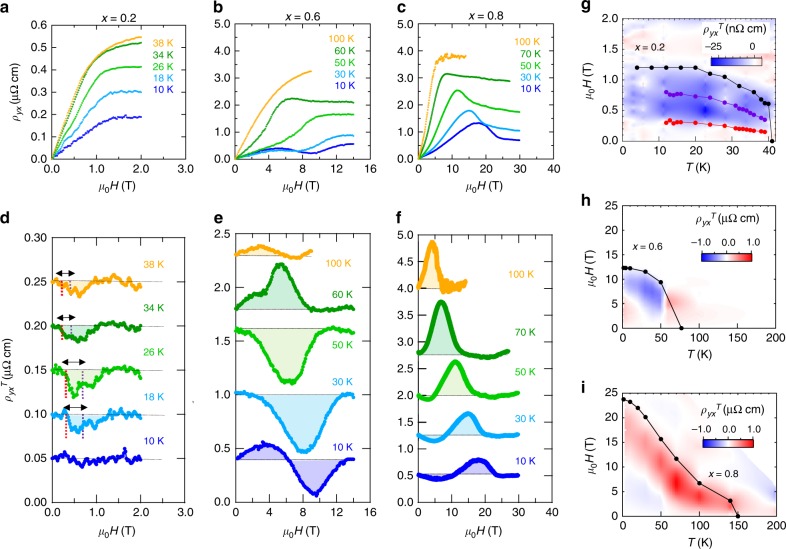
Topological Hall effect in MnSi_1−*x*_Ge_*x*_. **a**–**c** Hall resistivity $\rho _{yx}$ measured at various temperatures for *x* = 0.2 (**a**), *x* = 0.6 (**b**), and *x* = 0.8 (**c**). **d**–**f** Topological Hall resistivity $\rho _{yx}^{\mathrm{T}}$ estimated at various temperatures for *x* = 0.2 (**d**), *x* = 0.6 (**e**), and *x* = 0.8 (**f**). In panel **d**, the black double-headed arrows represent the *H*-region of skyrmion phase at each temperature; the red and purple dashed lines represent the lower- and higher-*H* boundaries, respectively. **g**–**i** Color maps of $\rho _{yx}^{\mathrm{T}}$ in the magnetic phase diagram for *x* = 0.2 (**g**), *x* = 0.6 (**h**), and *x* = 0.8 (**i**). The black dots and lines represent boundary between ferromagnetic state and helimagnetically ordered state. In panel **g**, the red and purple lines represent the lower- and higher-*H* boundaries for the skyrmion phase in *x* = 0.2. (See Supplementary Fig. 7)

In *x* = 0.6 and 0.8, we also detect characteristic SANS patterns under *H*: In addition to the intensity peaks at $\varphi = 0^\circ$, $180^\circ$, there appear peaks at $\varphi = \pm 70^\circ , \pm 110^\circ$ for $x = 0.6$ (Fig. 3h) and at $\varphi = \pm 90^\circ$ for $x = 0.8$ (Fig. 3i). We interpret these SANS patterns in terms of multiple-**q** structures. As for *x* = 0.6, Fig. 4 illustrates the possible multiple-**q** structure explaining the SANS result, i.e., the tetrahedral-4**q** state. One of the four **q**-vectors flips along the H-direction, as is the case of many other B20-type magnets^28^, and generates scattering intensity at $\varphi = 180^\circ$ ($0^\circ$), while the remaining three **q**-vectors produce intensities at $\varphi = \pm 70^\circ$ $(\pm 110^\circ)$ (Fig. 3k). As for *x* = 0.8, the observed intensity pattern is essentially identical to that of MnGe where the cubic-3**q** HL is realized^23^. In this case, one of the three **q**-vectors flips along the *H*-direction $\varphi = 180^\circ$ ($0^\circ$), while the other two orthogonal **q**-vectors generate scattering intensities at $\varphi = \pm 90^\circ$, as illustrated in Fig. 3l. Importantly, these 3**q** and 4**q** HL states dominates the whole magnetic-order phase below *H*_c_ shown in Fig. 1d, whereas the SkL state in the Si-rich region is generated by variation of *H* from the nearby helical/conical states (see Supplementary Fig. 7) as commonly observed in the SkL-hosting chiral magnets^28^.

Here we note that we cannot exclude a possibility of multi-domain state of the single-**q** helical structure on the basis of the SANS results alone. As demonstrated in the following section, however, such a scenario is incompatible with the observed large topological Hall effect, which is the hallmark of the formation of non-coplanar spin textures endowed with scalar spin chirality.

### High-magnetic-field measurements of topological Hall effect

Outcomes of these topological spin arrangements show up in the large topological Hall responses arising from their scalar spin chirality or skyrmion number^34^. The measured Hall resistivity $\rho _{yx}$ for $x = 0.2,\,0.6,\,0.8$ are summarized in Fig. 4a–c. We estimate the contributions from normal ($\propto H$) and anomalous ($\propto M$) Hall effects^35^ by reproducing $\rho _{yx}$ in the ferromagnetic (field-induced spin collinear) region with use of the fitting curve $\rho _{yx}^{{\mathrm{fit}}} = R_0H + R_{\mathrm{s}}\rho _{xx}^2M$, where $R_0$ and $R_{\mathrm{s}}$ are the normal and anomalous Hall coefficients, respectively (see Supplementary Fig. 3 for $M$ and Supplementary Fig. 10 for $\rho _{xx}$). Topological Hall effect (THE) arises as the deviation from the conventional contributions: $\rho _{yx}^{\mathrm{T}} = \rho _{yx} -$ $\rho _{yx}^{{\mathrm{fit}}}$. In MnSi_1−*x*_Ge_*x*_ (*x* = 0.2) hosting the SkL state, overall *H*-dependence of $\rho _{yx}$ obeys that of *M* (Supplementary Fig. 3), that suggests the dominant contribution from the conventional anomalous Hall effect (Fig. 4a). Besides, we identified negative $\rho _{yx}^{\mathrm{T}}$ in the intermediate *H*-region below *H*_c_ above 12 K (Fig. 4d). As seen in the color map of $\rho _{yx}^{\mathrm{T}}$ in Fig. 4g, $\rho _{yx}^{\mathrm{T}}$ reaches its maximum magnitude of $\left| {\rho _{yx}^{\mathrm{T}}} \right| = 30\,{\mathrm{n}}\Omega {\mathrm{cm}}$ near the center of SkL phase; and finite $\rho _{yx}^{\mathrm{T}}$ persists even in the conical phase, i.e., in the *H*-region between SkL and ferromagnetic states, implying that a part of skyrmions remain to subsist perhaps as disordered aggregation^4^.

In contrast, $\rho _{yx}$ in MnSi_1−*x*_Ge_*x*_ (*x* = 0.6 and 0.8) with HL states clearly indicate the large deviation from the conventional *M*-proportional profile, as shown in Fig. 4b, c. The estimated $\rho _{yx}^T$ shows complex behaviors with sign changes against *T* and *H* variations, as well as one-order-of-magnitude larger values than that in *x* = 0.2 (Fig. 4e, f, h, i.). The observed THE not only corroborate the formation of the topological multiple-**q** spin textures, but also exhibit different behaviors between tetrahedral-4**q** HL and cubic-3**q** HL. To be specific, MnSi_1−*x*_Ge_*x*_ with cubic-3**q** HL (*x* = 0.7–0.9 and MnGe^13^) share similarity in sign change of $\rho _{yx}^{\mathrm{T}}$: negative $\rho _{yx}^{\mathrm{T}}$ in the low-*T* and low-*H* region gives way to positive $\rho _{yx}^{\mathrm{T}}$ in the high-*T* and high-*H* region (Fig. 4f, i and Supplementary Fig. 11). The negative and positive $\rho _{yx}^{\mathrm{T}}$ may be attributed to static and fluctuating effects of emergent magnetic field^13,24,36^. As for *x* = 0.6 compound with tetrahedral-4**q** HL, the magnitude of $\rho _{yx}^{\mathrm{T}}$ is gigantic as well, while its *T*- and *H*-dependences are complicated to interpret (Fig. 4h). We note that such complex $\rho _{yx}^{\mathrm{T}}$-profiles with sign changes are also reported at the transitions between versatile topological spin structures, including 4**q**-HL, in SrFeO_3_^37,38^. By analogy with it, the sign change of $\rho _{yx}^{\mathrm{T}}$ at low temperatures in *x* = 0.6 (e.g., *T* *=* 10 K in Fig. 4e) may indicate a transition into different multiple-**q** states or the field-induced modification of the 4**q** structure, which remains an open question. We also note that there may exist robust or pinned excitations of spin hedgehogs even in the nominally ferromagnetic region^25^, which may imperil the validity of the present estimation of topological Hall effect. Hence, the magnitude and the sign changes of topological Hall resistivity may be difficult to quantitatively elucidate at the moment, while the presence of non-coplanar spin texture manifests itself by such anomalously large signals of topological Hall resistivity.

## Discussion

The present results not only on the SANS and LTEM but also on the topological Hall effects unveil the transitions among distinct topological spin textures, namely 2D SkL and two classes of 3D HLs in cubic chiral magnets MnSi_1−*x*_Ge_*x*_. Compared with the case of SrFeO_3_ where magnetic domains with different helicity and vorticity degrees of freedom should coexist due to the centrosymmetric crystal structure^38^, a point of uniqueness in MnSi_1−*x*_Ge_*x*_ is ascribed to the fixed helicity and vorticity over the whole chiral lattice. In addition, the transitions between different topological spin textures can be realized simply by controlling lattice constant; therefore, it would be possible to switch the topology of spin textures by application of small pressure or strain, once a composition *x* is tuned to the transition points (*x* ~ 0.3 and 0.7). Given that pressure is a fundamental variable that controls the properties of materials by changing inter-atomic distance or electron transfer interaction^39^, the impact of pressure on topological spin textures deserves further investigations in a wide range of materials in the light of the exploration of novel spin textures and emergent electrodynamics.

## Methods

### Sample preparation

Polycrystalline samples of MnSi_1−*x*_Ge_*x*_ were prepared by the high-pressure synthesis technique. Mn, Si, and Ge were first mixed with stoichiometric ratio and then melted in an arc furnace under an argon atmosphere. Afterwards, it was heated at 1073 K for 1 h under 5.5–6.0 GPa with a cubic-anvil-type high-pressure apparatus. Powder x-ray analyses confirmed B20-type crystal structure (*P*2_1_3).

### Magnetic and transport property measurements

Magnetization was measured either by using ACMS option with Physical Property Measurement System (PPMS) or by DC option with Magnetic Property Measurement System (MPMS). Magneto-resistivity and Hall-resistivity up to 14 T were measured by using AC-transport option with PPMS. Magnetic field was applied perpendicular to electrical current. Higher-field measurements of magnetization and Hall resistivity were performed utilizing non-destructive pulse magnets energized by capacitor banks and a flywheel DC generator installed at International MegaGauss Science Laboratory of Institute for Solid State Physics (ISSP), University of Tokyo, Japan, respectively. The high-field magnetization was measured up to 56 T by the conventional induction method, using coaxial pickup coils. The high-field resistivity was measured up to 30T using the long (~1s) field pulse with the AC four-wires method employing a numerical phase detection technique with a sampling rate of 200,000 data points per second and an excitation current of 10 kHz and 200 mA_p-p_.

### Lorentz TEM observations

Lorentz TEM observations for a (001) MnSi_0.8_Ge_0.2_ thin plate were performed using a multifunctional transmission electron microscope (JEM2800, JEOL) equipped with double-tilt helium cooling holder (Gatan ULTDT). The thin plate was prepared by an Ar^+^ milling process after mechanical polishing of the bulk sample.

### Neutron scattering

Neutron scattering experiments were performed at small- and wide-angle neutron scattering instrument (TAIKAN) built at BL15 of Materials and Life Science Experimental Facility (MLF) in Japan Proton Accelerator Research Complex (J-PARC)^40^. A powder sample of MnSi_1−*x*_Ge_*x*_ was packed in an aluminum container filled by He gas, and installed in a cryomagnet. The weight of the powder sample was $0.450\,{\mathrm{g}}\left( {x = 0.2} \right)$, $0.719\,{\mathrm{g}}\left( {x = 0.4} \right)$, $1.059\,{\mathrm{g}}\left( {x = 0.6} \right)$, and $0.751\,{\mathrm{g}}\left( {x = 0.8} \right)$. Magnetic field was applied perpendicular to the incident neutron beam. The diffracted neutron beam with the wavelength of $0.5 < \lambda < 7.8$ Å was collected by four detector banks of small-, middle-, and high-angle and backward detector banks, and analysed by using time-of-flight (TOF) method.

### Band structure calculations

Electronic structure calculations were performed using the plane-wave basis set with the projector augmented wave (PAW) scheme^41^ as implemented in the Vienna Ab initio Simulation Package (VASP)^42,43^. The Perdew-Burke-Ernzerhof (PBE) exchange-correlation functional^44^ and a cutoff energy of 500 eV were used. Experimental crystal structures^45,46^ were adopted and the lattice constants were changed by fixing the fractional coordinates of the atoms. Dzyaloshinskii-Moriya interactions were evaluated using a spin current formalism^29,47^. To calculate the energy of the spiral spin structures, a generalized Bloch theorem was employed^48^. The effect of strong correlation was discussed using the DFT + U method^49^.

## Supplementary information

Supplementary Information

Source Data

## Data Availability

The data sets generated during and/or analysed during the current study are available from the corresponding author on reasonable request.
