# Supplementary material for: Topological transitions among skyrmion- and hedgehog-lattice states in cubic chiral magnets
Source: Nat Commun. 2019 Mar 5;10:1059. doi: 10.1038/s41467-019-08985-6 (PMC6401095; doi:10.1038/s41467-019-08985-6)
Supplement: Supplementary file 1 — Supplementary Information [file 41467_2019_8985_MOESM1_ESM.pdf]

Supplementary Information for

Topological transitions among skyrmion- and hedgehog-lattice states in cubic chiral magnets

Y. Fujishiro[1, *], N. Kanazawa[1, *], T. Nakajima[2], X. Z. Yu[2], K. Ohishi[3], Y. Kawamura[3], K. Kakurai[2,3], T. Arima[2,4], H. Mitamura[5], A. Miyake[5], K. Akiba[5], M. Tokunaga[5], A. Matsuo[5], K. Kindo[5], T. Koretsune[6], R. Arita[1,2] and Y. Tokura[1,2]

[1] *Department of Applied Physics, The University of Tokyo, Bunkyo-ku, Tokyo 113-8656, Japan*

[2] *RIKEN Center for Emergent Matter Science (CEMS), Wako, Saitama 351-0198, Japan*

[3] *Neutron Science and Technology Center, Comprehensive Research Organization for Science and Society (CROSS), Tokai, Naka, Ibaraki 319-1106, Japan*

[4] *Department of Advanced Materials Science, The University of Tokyo, Kashiwa, Chiba 277-8561, Japan*

[5] *The Institute of Solid State Physics (ISSP), The University of Tokyo, Kashiwa, Chiba 277-8581, Japan*

[6] *Department of Physics, Tohoku University, Aoba-ku, Sendai, Miyagi 980-8578, Japan*

* To whom correspondence should be addressed.

E-mail: fujishiro@cmr.t.u-tokyo.ac.jp and kanazawa@ap.t.u-tokyo.ac.jp

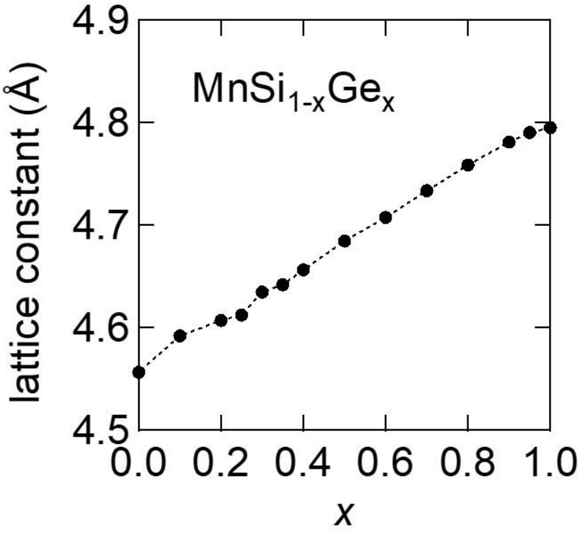

Supplementary Figure 1 | Variation of lattice constant in $MnSi_{1-x}Ge_x$. The lattice constant is determined from powder x-ray diffraction patterns. The value of MnSi ($x = 0$) is reproduced from Supplementary Ref. 1.

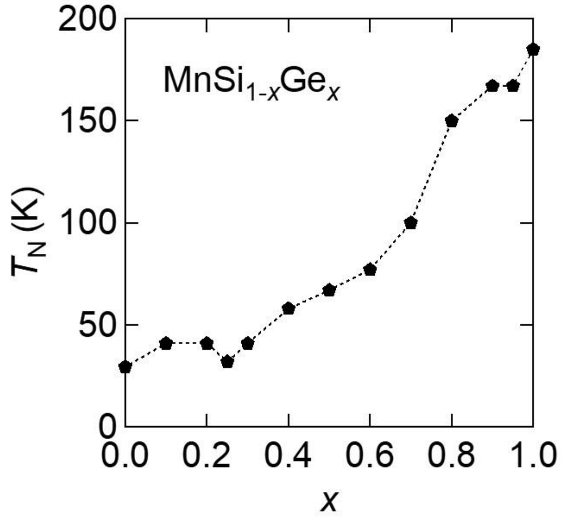

Supplementary Figure 2 | Variation of magnetic transition temperature T_N in $MnSi_{1-x}Ge_x$.

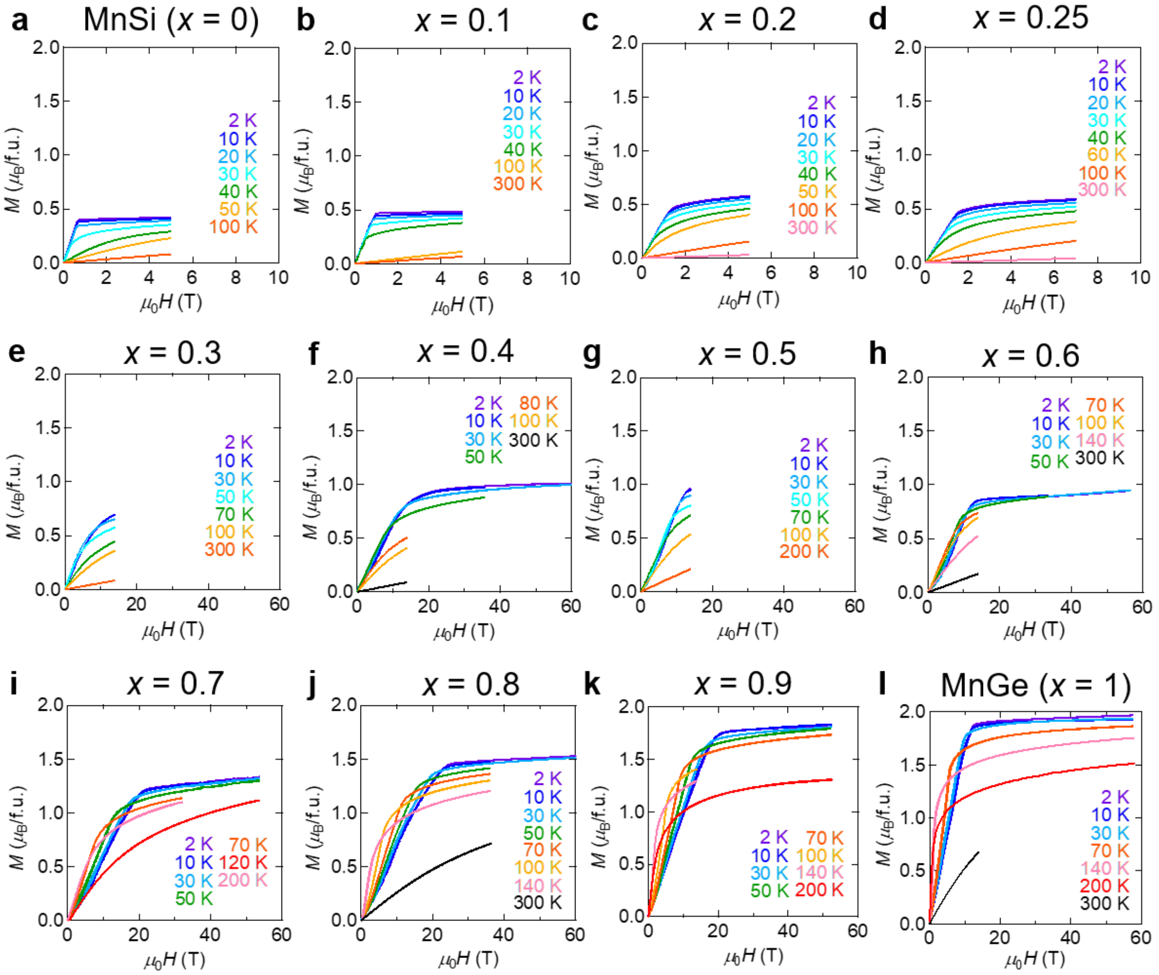

Supplementary Figure 3 | Magnetization curves at various temperatures in $MnSi_{1-x}Ge_x$.

a-l, The magnetization curve shows an inflection corresponding to the ferromagnetic transition at the critical magnetic field H_c. Saturation magnetization M_s was defined as M at H_c at the lowest temperature $T = 2$ K.

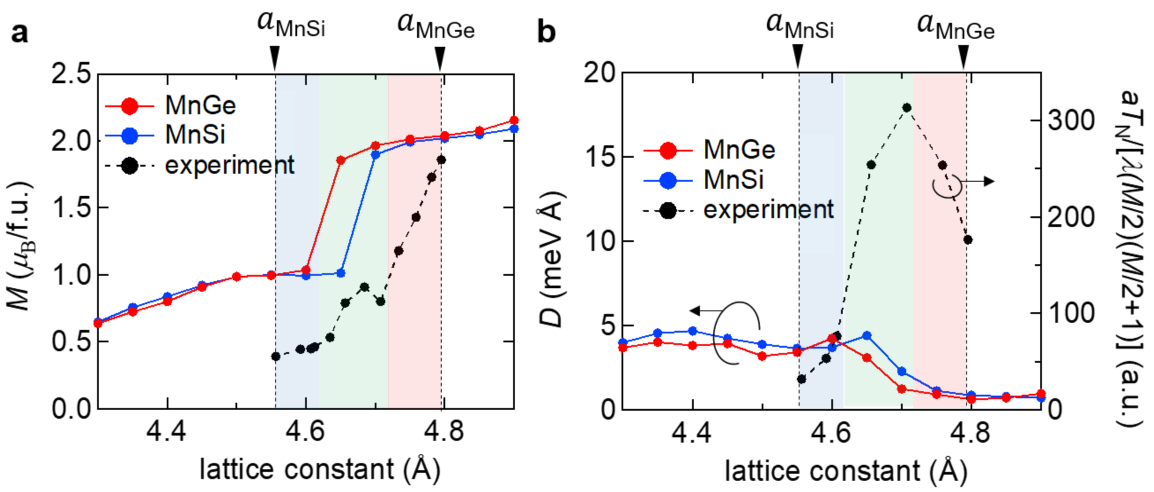

Supplementary Figure 4 | Comparisons between theoretical and experimental estimations of magnetization and strength of Dzyaloshinskii-Moriya interaction with varying lattice constant. a, b, Estimated magnetization M (**a**) and Dzyaloshinskii-Moriya interaction (DMI) D (**b**) by the band structure calculations with virtually changing the lattice constants a of MnGe (red dots and lines) and MnSi (blue dots and lines). The black dots with dashed lines represent the experimental values of the saturation magnetization M_s at 2 K (**a**) and an indicator of the DMI strength, $aT_N/[\lambda(M_s/2)(M_s/2+1)]$, under the assumption that the mangetic modulation period λ is given by the ratio of the ferromagnetic exchange interaction J to D ($\lambda = aJ/D$) (**b**) in $MnSi_{1-x}Ge_x$. For the experimental estimation of DMI[2], we employed the following relation: $T_N = \frac{z_0 JS(S+1)}{3k_B}$ deduced from the Heisenberg Hamiltonian $\mathcal{H} = -\sum_{<ij>} J\mathbf{S}_i \cdot \mathbf{S}_j$ with the number of the nearest neighbor atoms z_0. The blue, green and red regions correspond to the experimentally-revealed magnetic phases of skyrmion lattice (SkL), tetrahedral-4**q** hedgehog lattice (HL), and cubic-3**q** HL states in $MnSi_{1-x}Ge_x$, respectively. In panel **a**, the overall tendencies of M are consistent with each other: with increasing the lattice constant a, M shows gradual increase in a = 4.3–4.5 Å (theory), a = 4.56–4.61 Å (experiment); plateau structure in a = 4.5–4.6 Å (theory), a = 4.63–4.71 Å (experiment); steep increase in a = 4.6–4.7 Å (theory), a = 4.73–4.80 Å (experiment), despite the quantitative mismatch in a. As

shown in **b**, in contrast, the theoretical and experimental DMI show completely opposite behaviours to each other: decreasing (theory) and increasing (experiment) profiles with increasing the lattice constant *a*. This suggests that some other dominant mechanism(s) may rule the magnetic modulation length in the Ge-rich region, as discussed in the main text.

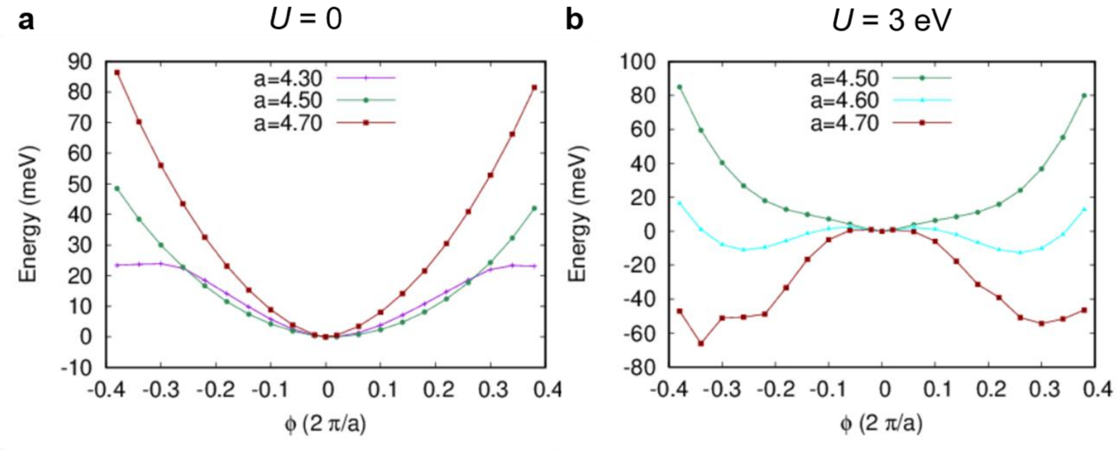

Supplementary Figure 5 | Variation of calculated total energy for MnGe with/without on-site Coulomb repulsion *U* with varying lattice constant *a* and helical spin wavenumber *ϕ*. **a, b,** Helical-modulation-wavenumber ϕ (in the unit of $2\pi/a$) dependence of total energy estimated by the band structure calculations with $U = 0$ eV (**a**) and $U = 3$ eV (**b**). The energy minima are always realized around $\phi = 0$ of the ferromagnetic state when $U = 0$ eV (**a**). With taking into account $U = 3$ eV, the short-period helical state becomes more stable than the ferromagnetic state when *a* is larger than 4.60 Å, which may explain formation of the short-period hedgehog-lattice states in $MnSi_{1-x}Ge_x$.

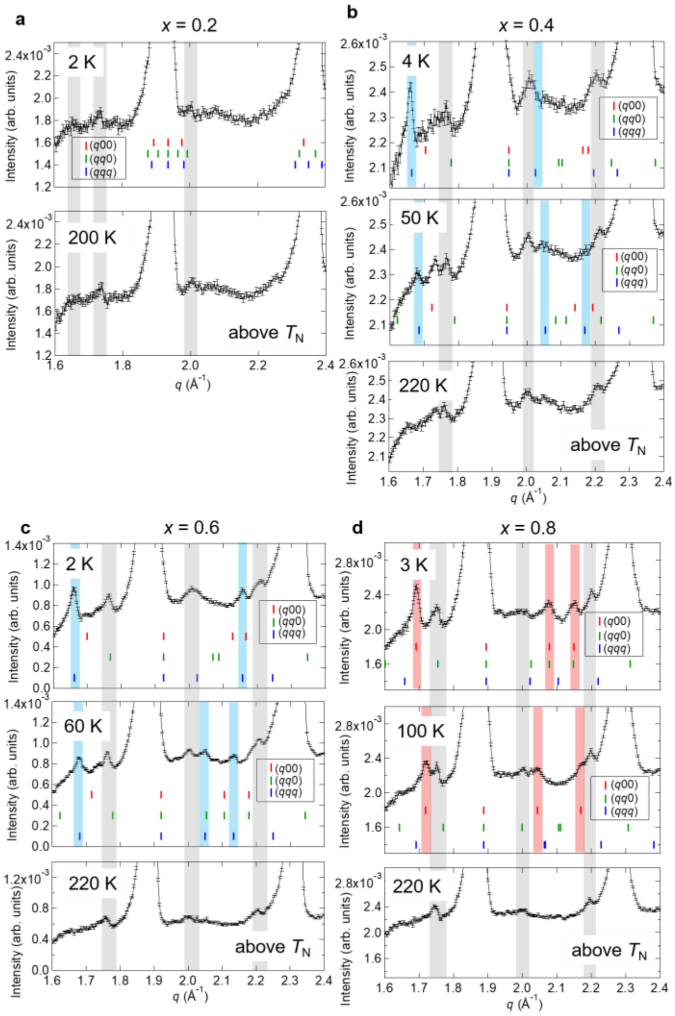

Supplementary Figure 6 | Wide-angle neutron scattering on $MnSi_{1-x}Ge_x$ at zero magnetic field. a-d, Powder neutron diffraction patterns obtained by using the wide-angle detector for x = 0.2 (**a**), 0.4 (**b**), 0.6 (**c**), 0.8 (**d**). The small colour bars indicate the position of estimated satellite peaks $\mathbf{q}_s = \mathbf{q}_n + \mathbf{q}$, which vary depending on the relative directions of nuclear and magnetic modulations vectors $\mathbf{q}_n$ and $\mathbf{q}$ (Supplementary Ref. 4). Except for the case of $x = 0.2$, where the magnetic satellite peaks are buried in the nuclear reflections, we could assign the direction of $\mathbf{q}$ fixed by magnetic anisotropy at zero magnetic field: <111> directions for $x = 0.4$ and 0.6; <100> directions for x = 0.8. The diffraction peaks shaded by gray are nuclear reflections from impurity because they appear above the magnetic transition temperatures T_N. The error bars represent statistical error of one standard deviation.

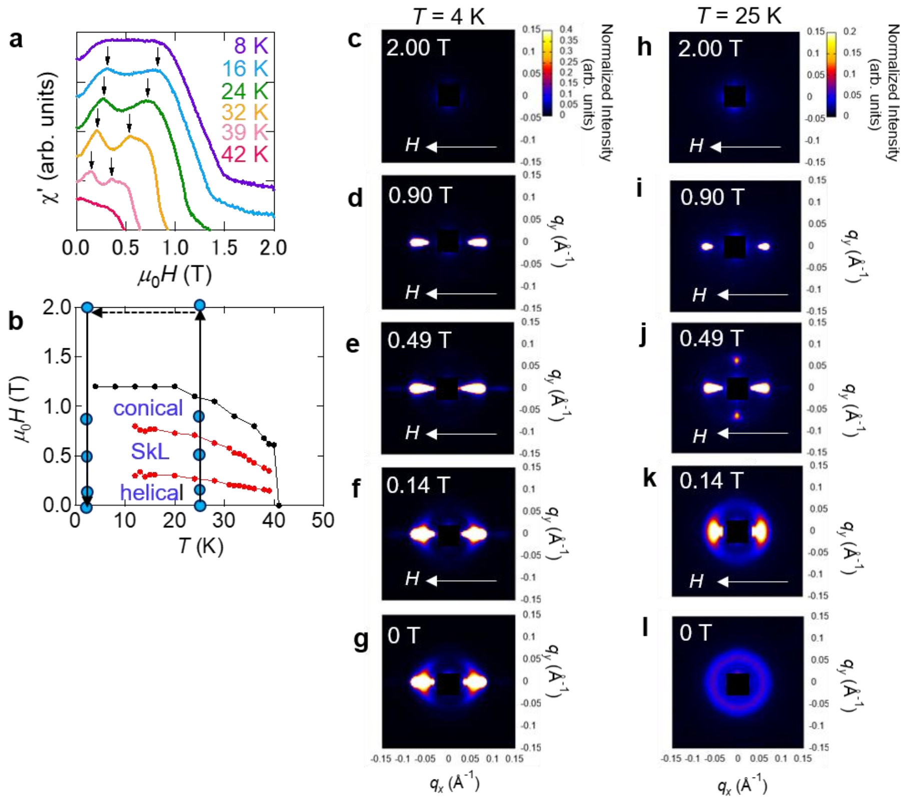

Supplementary Figure 7 | Magnetic phase diagram and small-angle neutron scattering (SANS) patterns for $MnSi_{1-x}Ge_x$ (x = 0.2). a, Magnetic field dependence of the real part of ac susceptibility measured at various temperatures for x = 0.2. The data are shifted by constant values for clarity. The observed two kink structures, indicated by black arrows for each temperature, correspond to the transition from helical to skyrmion phase and from skyrmion to conical phase, respectively[3]. **b,** Magnetic phase diagram of $MnSi_{1-x}Ge_x$ (x = 0.2), where the red lines represent the skyrmion lattice (SkL) phase boundaries determined from ac susceptibility as shown in **a**. The black line represents the ferromagnetic transition boundary. The blue markers and black arrows show the points and process of the SANS measurement. **c-l,** SANS patterns at various magnetic fields H at 4 K (**c-g**) and at 25 K (**h-l**). The H-direction is indicated by the white arrow in **c-f**, **h-k**. The elongated shape of the intensities for helical/conical state may originate from scatterings from magnetic impurity, which are observed at $q_{\text{impurity}} = 0.0367\ \text{Å}^{-1}$ (data not shown).

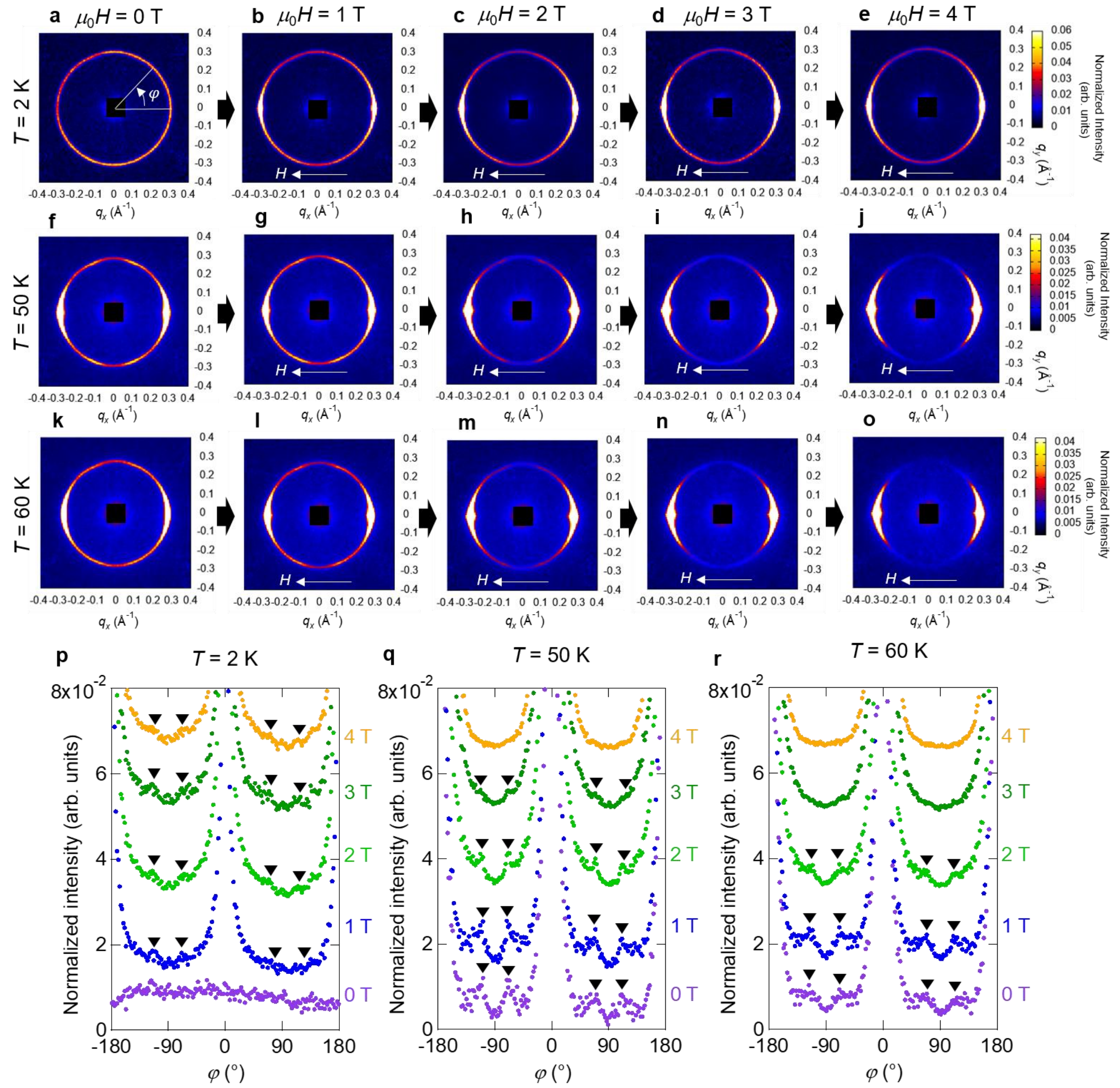

Supplementary Figure 8 | Small-angle neutron scattering (SANS) experiment under magnetic field (H) for x = 0.6. a-o, Magnetic-field (H) dependence of SANS intensity patterns of x = 0.6 measured at T = 2 K (**a-e**), T = 50 K (**f-j**), T = 60 K (**k-o**). The thick black arrows represent the measurement sequences which correspond to the alphabetical order of the panel. **p-r**, Azimuth-angle φ dependence of the SANS intensity shown in panel **a-o**. The definition of φ is shown in panel **a**. The black triangles highlight the intensity peaks observed at φ = ± 70 ° and ± 110 °. For T = 50 K and 60 K, these characteristic intensity peaks appear even at H = 0, which is possibly caused by the rotation of multiple-**q** state by H. While the directions of **q**-

vectors are pinned along <111> crystal axes at $H = 0$ T, they are rotated by H in a way that one of the **q**-vectors is flipped along H-direction and the other **q**-vectors are correspondingly rotated as being dragged by the flipped **q**-vector. Once the rotation of the multiple-**q** state occurs, the **q**-vectors do not return to their originally pinned directions and stay in the rotated directions even after removing H, as long as the temperature is not raised much above the magnetic transition temperature (Supplementary Ref. 5 & 6). Since the SANS measurement at $T = 50$ K and 60 K are performed after the H-scan measurement at $T = 2$ K, the q-vectors are already in the rotated directions, producing the characteristic intensity peaks even at $H = 0$ T. Furthermore, the partial alignment of the sample powder grains, being dragged by the alignment of the easy axes of magnetization (magnetic modulation directions), can occur upon application of H, which also contributes to the appearance of the characteristic SANS intensity peaks observed at $T = 50$ K and 60 K even at $H = 0$ T.

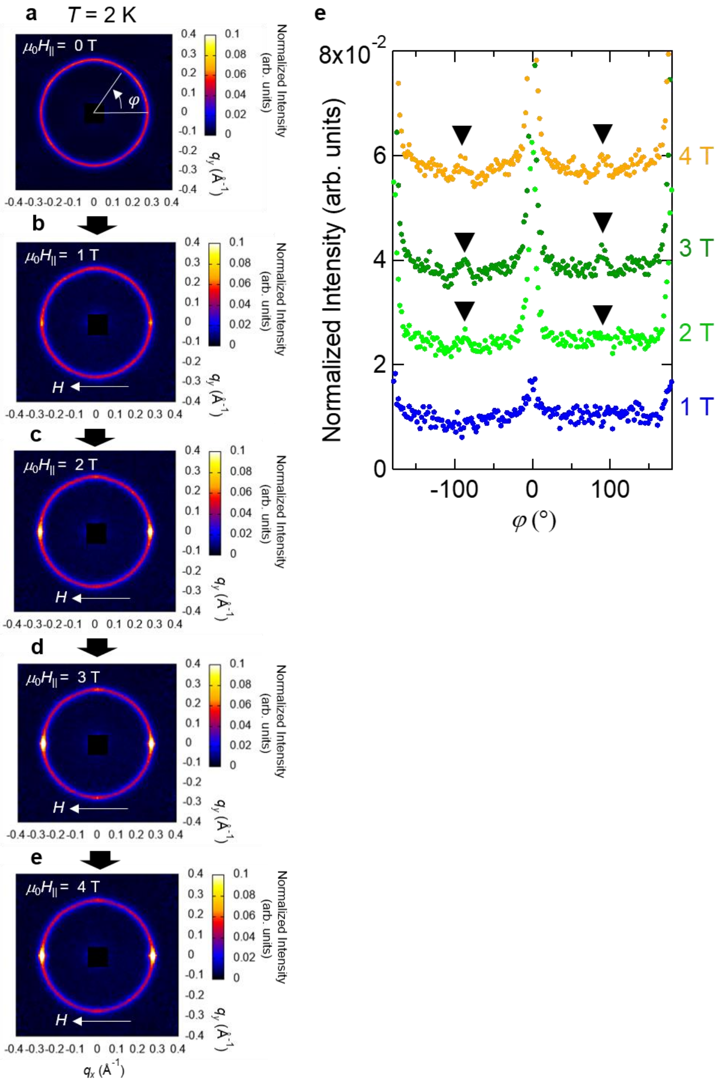

Supplementary Figure 9 | Small-angle neutron scattering (SANS) experiment under magnetic field (*H*) for *x* = 0.8. a-e, SANS intensity patterns of $x = 0.8$ measured at $T = 2$ K under H ($\mu_0 H = 0$ T - 4 T). The black arrows represent the measurement sequences. **f**, Azimuth-angle φ dependence of the SANS intensity shown in panel **b-e**. The definition of φ is shown in panel **a**. The black triangles highlight the intensity peaks observed at $\varphi = \pm 90^\circ$.

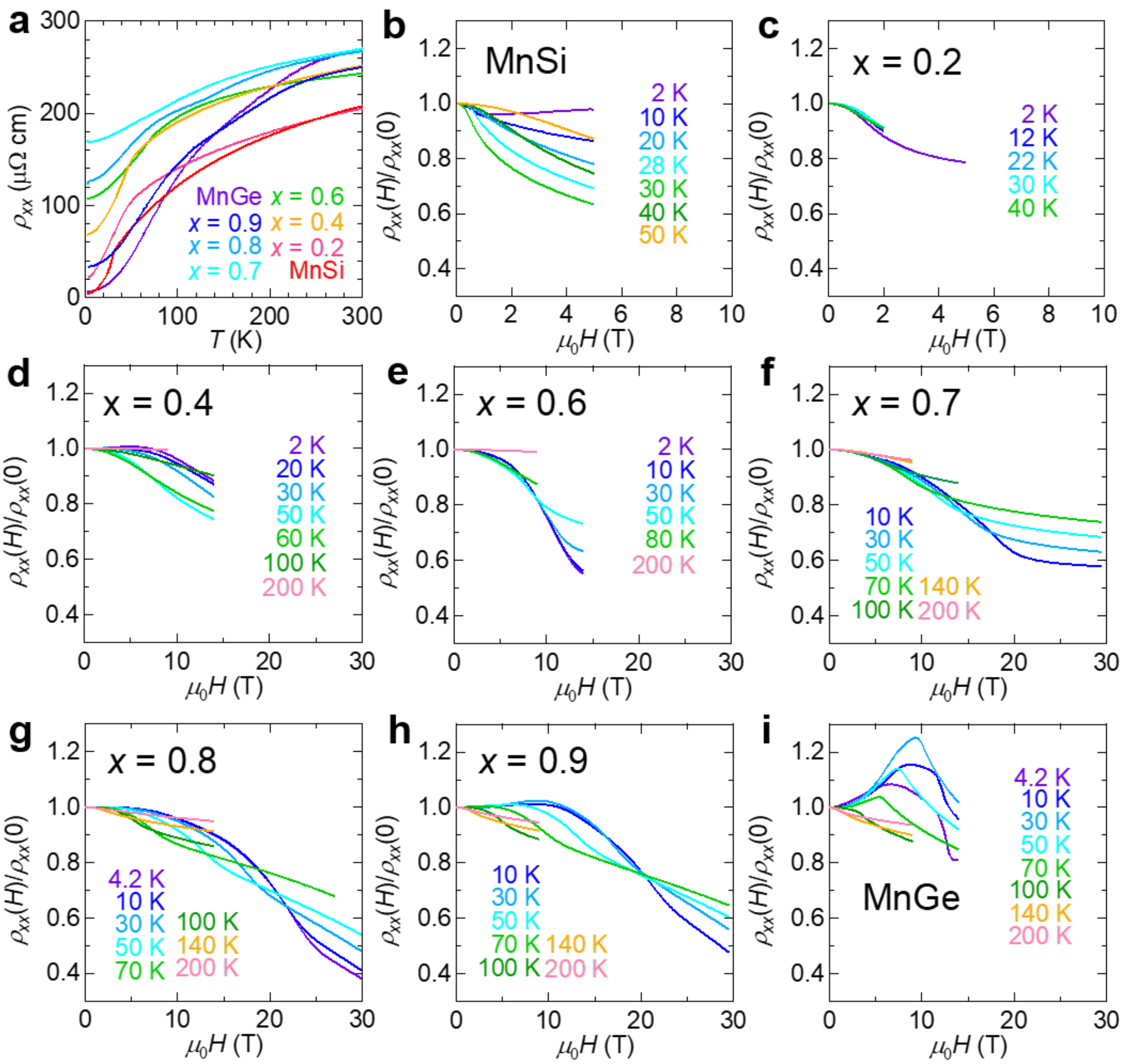

Supplementary Figure 10 | Magneto-resistivity in $MnSi_{1-x}Ge_x$ (x = 0, 0.2, 0.4, 0.6, 0.7, 0.8, 0.9 and 1). a, Temperature dependence of resistivity. **b-i**, Normalized magneto-resistivity ρ_{xx} measured at various temperatures.

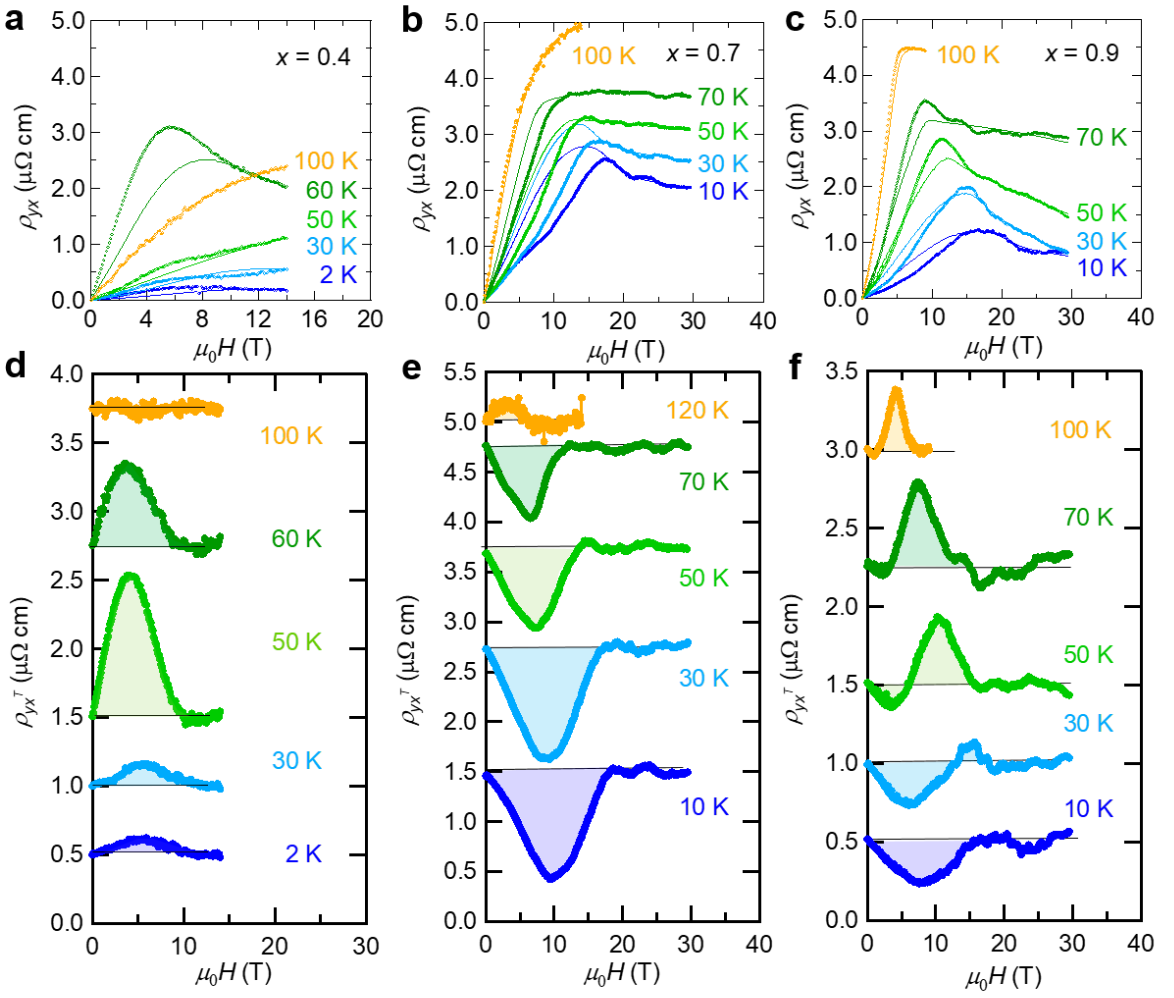

Supplementary Figure 11 | Hall resistivity in $MnSi_{1-x}Ge_x$ (x = 0.4, 0.7 and 0.9). a-c, Hall resistivity ρ_{yx} measured at various temperatures (solid dots) along with the fitting curves $\rho_{yx}^{\mathrm{fit}} = R_0H + R_s\rho_{xx}^2M$ (solid lines). **d-f,** Estimated topological Hall resistivity ρ_{yx}^{T}. The data are shifted by constant values for clarity.

Supplementary reference

1. Ishikawa, Y. et al. Magnetic Excitations in the Weak Itinerant Ferromagnet MnSi. *Phys. Rev. B* **16**, 4956–4970 (1977).
2. Kanazawa, N., Shibata, K. & Tokura. Y. Variation of Spin-orbit coupling and related properties in skyrmionic system $Mn_{1-x}Fe_xGe$. *New J. Phys.* **18**, 045006 (2016).
3. Bauer, A. & Pfleiderer, C. Magnetic phase diagram of MnSi inferred from magnetization and ac susceptibility. *Phys. Rev. B* **85**, 214418 (2012).
4. Kanazawa, N. et al. Large Topological Hall Effect in a Short-Period Helimagnet MnGe. *Phys. Rev. Lett.* **106**, 156603 (2011)
5. Grigoriev, S. V. et al. Magnetic structure of $Fe_{1-x}Co_xSi$ in a magnetic field studied via small-angle polarized neutron diffraction. *Phys. Rev. B* **76**, 224424 (2007).
6. Kanazawa, N. et al. Possible skyrmion-lattice ground state in B20 chiral-lattice magnet MnGe as seen via small-angle neutron scattering. *Phys. Rev. B* **86**, 134425 (2012)
